# Dental Law—Holland

**Published:** 1895-10

**Authors:** 


### Dental Law.—Holland.

LAW REGULATING THE CONDITION FOR OBTAINING QUALIFICATION OF DENTIST,* OF DEC. 25TH, 1878, ALTERED DEC. 12TH, 1892.

ARTICLE 8.--The title of dentist, allows the practice of dentistry, by which is understood the local treatment of disease of the teeth, their alveoli and gums. This title can be obtained upon successfully passing a practical examination, in which sufficient evidence must be given of the practical knowledge of operative dentistry and inserting artificial teeth and plates.

ARTICLE 9.—Only those who have successfully passed the theoretical examination can be considered for the practical examination. This examination includes:

(*a*) Anatomy of the teeth, their aveoli and gums.

(*b*) Physiology of these parts.

(*c*) Hygiene, pathology of and medicaments for these parts, in which is included the capability of distinguishing diseases of the teeth, their aveolar processes and gums, of which the cause is general or resides in other parts.

(*d*) Materia medica, and knowledge of prescription writing, as far as necessary to prescribe local medicaments for diseases above mentioned.

The following is an extract of the by-laws, etc.,

*Dutch: "Tandmeister." German: "Zahnmeister." Certainly a title far inferior to the American D. D. S.

The title of "Tandarts," German "Zahnartz," is however allowed in Holland and is now mostly adopted.

Those, who have after examination obtained the right to practice dentistry in another country, or in one of the Dutch colonies, may be partially or entirely excused from the theoretical examination and from the preceding examinations. The Government reserves the right, after having been advised by the Government University, to decide which certificates or diplomas excuse the holders from the examination, either entirely or partially.

Excused from an examination in branches under letters (*a*) and (*b*), are:

Those who have attained a diploma as doctor of medicine of one of the Holland Universities, also those who have passed second physical examination.*

To come in consideration for the theoretical examination (leiste natum kundig) for dentist, the first physical examination must have been passed, or permission obtained from the professors of the University to be examined for it.†

The theoretical examination is conducted by members of the medical faculty of the Netherland Government Universities, fee $10.00.

Practical examination $1[illegible].00.

In case of refusal the next examination is free of charge, a third examination $10.00 each.

The committee for examination consists of the president, and members, place-taking members, and secretary, and are appointed by the Government each year.

Examinations are held in public, except those on the sick-bed, to which others than the candidate may be present, by permission of the examining committee only.

Those who pass receive a diploma which gives the right to practice dentistry.

Those passed must, before they are admitted, swear and solemnly declare to practice dentistry according to the constituted law, to their best knowledge and ability, and not to make public

*This is one of the examinations of our universities, and rather severe.

†The examination in Holland is always conducted by a committee appointed by the Government, in which the teaching professors have no part.

to any one, what has, in confidence, been communicated to them, or has come to their knowledge, unless demanded by the law as witness. "So help me God Almighty." (I promise it.)

Examination is held twice at least, each year.

The Minister of Interior decides the place and time for the committee to assemble.

Examination is conducted in the Dutch language only.

The money paid by the candidate goes to the Government, which pays the examining committee a regular fee.

The entire regulation of the examination is constituted by the Government.

A dentist is allowed to treat diseases of the teeth, alveoli and gums, by prescribing medicaments for those parts.

General anæsthesia, prescribing of systemic remedies and furnishing of medicine is forbidden.

Before engaging in practice the diploma must be revised by the medical inspector of the province in which one wishes to locate--and must furnish a legal proof of locating.

On moving, the inspector must be again notified, and certificate issued by him returned.

The Lord Mayor of the town in which one locates as dentist must also be notified officially.

The same regulations hold good for temporary residing at a place.

Practicing without diploma or infringing the dental law is punished with from one to six months imprisonment *and* fine of from $10.00 to $40.00, together or separately.

Practicing without having diploma revised or registered is punished with a fine of from $4.00 to $40.00.

In conclusion allow me to say, that if that gentleman has passed the examination under the new law of '92, he likely will be well posted on those branches, as I know the examining committee are very strict and honest, but if he is an "old timer," I should think that his theoretical knowledge would be very limited.

The practical part receives so little attention in Holland, that the "old time" dentists know little more than extracting teeth and making plates of rubber and probably of gold.

